# Supplementary material for: A haplotype-resolved chromosome-level genome assembly of Urochloa decumbens cv. Basilisk resolves its allopolyploid ancestry and composition
Source: G3 (Bethesda). 2025 Jan 24;15(4):jkaf005. doi: 10.1093/g3journal/jkaf005 (PMC12005165; doi:10.1093/g3journal/jkaf005)
Supplement: jkaf005_Supplementary_Data [file jkaf005_supplementary_data.zip › Supplemental_Figure_Legends_G3-2024-405562.docx]

**SUPPLEMENTARY FIGURE LEGENDS**

*A haplotype-resolved chromosome-level genome assembly of Urochloa decumbens cv. Basilisk resolves its allopolyploid ancestry and composition*

Figure S1: Kmer spectra supported the assembly accurately captures the sequence information in the HiFi reads.

Figure S2: Coverage (read depth) following the alignment of reads from (a) U. decumbens (blue) and (b) U. ruziziensis (orange) evidence both aligned almost in the same target regions.
