# Supplementary figures and images for: A haplotype-resolved chromosome-level genome assembly of Urochloa decumbens cv. Basilisk resolves its allopolyploid ancestry and composition
Source: G3 (Bethesda). 2025 Jan 24;15(4):jkaf005. doi: 10.1093/g3journal/jkaf005 (PMC12005165; doi:10.1093/g3journal/jkaf005)

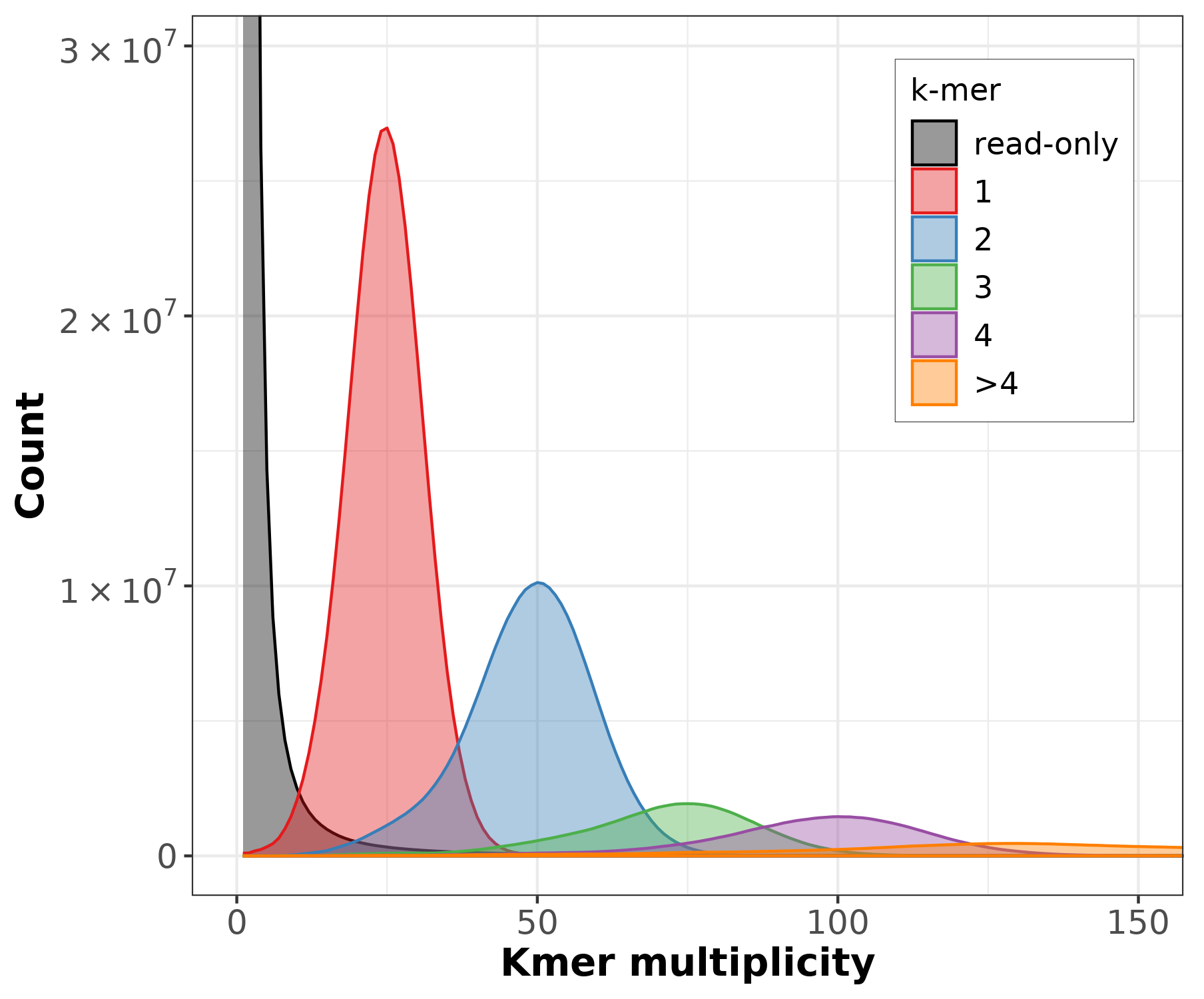

Supplement: jkaf005_Supplementary_Data [file jkaf005_supplementary_data.zip › Figure_S1_G3-2024-405562.png]

A

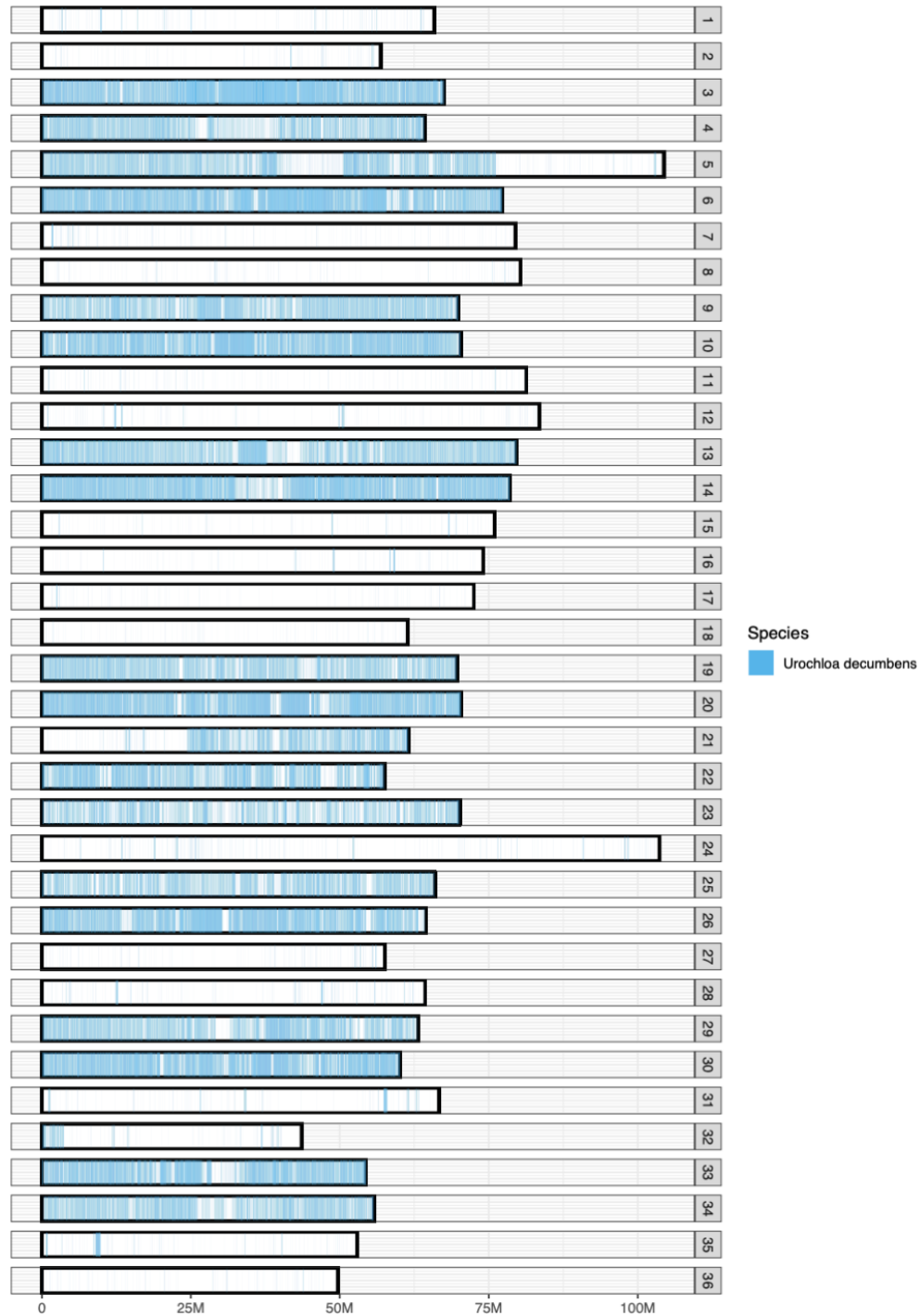

B

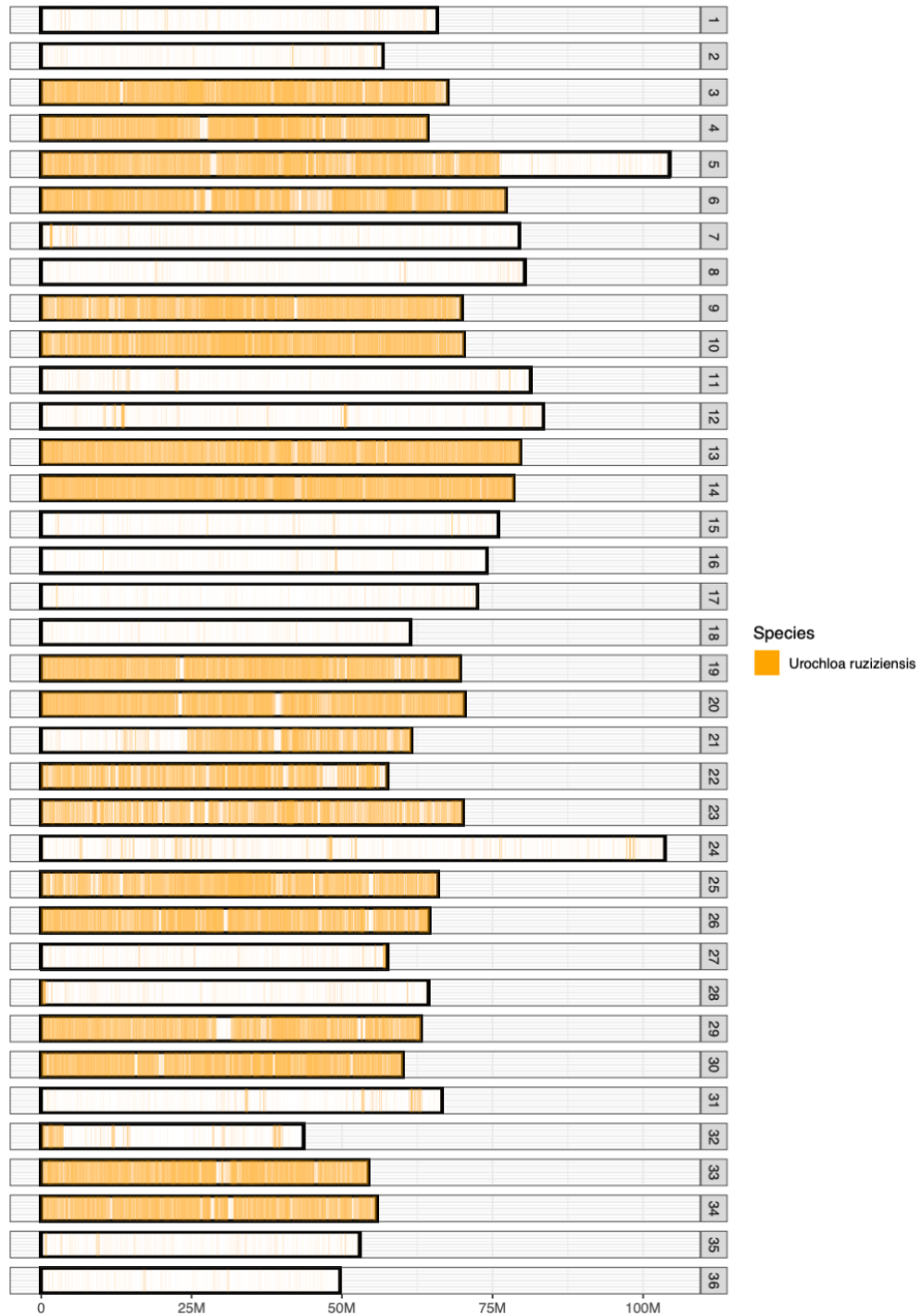

Supplement: jkaf005_Supplementary_Data [file jkaf005_supplementary_data.zip › Figure_S2_G3-2024-405562.pdf]
